# The Protease Inhibitor Amprenavir Protects against Pepsin-Induced Esophageal Epithelial Barrier Disruption and Cancer-Associated Changes

**DOI:** 10.3390/ijms24076765

**Published:** 2023-04-05

**Authors:** Simon Blaine-Sauer, Tina L. Samuels, Ke Yan, Nikki Johnston

**Affiliations:** 1 Department of Otolaryngology and Communication Science, Medical College of Wisconsin, Milwaukee, WI 53226, USA; sblaine@mcw.edu (S.B.-S.);; 2 Department of Pediatrics Quantitative Health Sciences, Medical College of Wisconsin, Milwaukee, WI 53226, USA; 3 Department of Microbiology and Immunology, Medical College of Wisconsin, Milwaukee, WI 53226, USA

**Keywords:** gastroesophageal reflux disease, antireflux therapeutics, pepsin, amprenavir, fosamprenavir, protease inhibitors, Barrett’s esophagus, esophageal adenocarcinoma

## Abstract

Gastroesophageal reflux disease (GERD) significantly impacts patient quality of life and is a major risk factor for the development of Barrett’s esophagus (BE) and esophageal adenocarcinoma (EAC). Proton pump inhibitors (PPIs) are the standard-of-care for GERD and are among the most prescribed drugs in the world, but do not protect against nonacid components of reflux such as pepsin, or prevent reflux-associated carcinogenesis. We recently identified an HIV protease inhibitor amprenavir that inhibits pepsin and demonstrated the antireflux therapeutic potential of its prodrug fosamprenavir in a mouse model of laryngopharyngeal reflux. In this study, we assessed the capacity of amprenavir to protect against esophageal epithelial barrier disruption in vitro and related molecular events, E-cadherin cleavage, and matrix metalloproteinase induction, which are associated with GERD severity and esophageal cancer. Herein, weakly acidified pepsin (though not acid alone) caused cell dissociation accompanied by regulated intramembrane proteolysis of E-cadherin. Soluble E-cadherin responsive matrix metalloproteinases (MMPs) were transcriptionally upregulated 24 h post-treatment. Amprenavir, at serum concentrations achievable given the manufacturer-recommended dose of fosamprenavir, protected against pepsin-induced cell dissociation, E-cadherin cleavage, and MMP induction. These results support a potential therapeutic role for amprenavir in GERD recalcitrant to PPI therapy and for preventing GERD-associated neoplastic changes.

## 1. Introduction

Gastroesophageal reflux disease (GERD) affects up to 25% of adults in high-income countries and has significant adverse effects on patient quality of life [1,2,3]. Typical symptoms of GERD include heartburn and regurgitation, and concomitant extraesophageal symptoms such as cough and hoarseness are common [1,4,5]. Patients with GERD are at increased risk for developing complications such as esophageal strictures and Barrett’s esophagus (BE; columnar metaplasia of the esophageal epithelium) and have a 43.5-fold greater risk of developing esophageal adenocarcinoma (EAC) [1,6,7,8].

Proton pump inhibitors (PPIs) are the first-line therapy against GERD and are among the most prescribed drugs in the world. Studies conducted in developed nations have reported PPI use by up to 30% of all adults and greater than 50% of those over 65 with inappropriate prescriptions, and over-the-counter availability contributing to excessive use [9,10,11,12,13,14,15]. The global economic burden of PPIs exceeds over $25 billion annually [16,17]. Despite the widespread use of PPIs, the global incidence of EAC has rapidly risen in recent decades and at current rates will reach 141,300 new cases in 2040 [18,19,20,21]. A recent meta-analysis to evaluate the chemopreventive benefit of PPIs found no significant reduction in EAC risk [22]. Instead, high-quality observational trials have demonstrated that long-term high adherence to PPIs may increase EAC risk [23,24]. The association of PPI use with EAC may be due to symptom masking or the carcinogenic potential of the drugs themselves as supported by the association of their use with increased incidence of digestive tract cancers generally, and pancreatic, gastric, and liver cancers specifically [25]. Physicians and patients have grown increasingly concerned regarding the health risks of long-term PPI therapy prompting the investigation of alternatives [26,27].

PPIs reduce the secretion of acid by parietal cells in the stomach, thereby reducing the acidity of the refluxate. PPIs do not reduce the number of reflux events [28,29] and do not address damage by nonacid reflux components such as pepsin and bile salts [30,31]. Instead, studies indicate that PPIs may increase the concentration and toxicity of nonacid constituents of refluxate [30,32]. In the context of weakly to nonacidic refluxate such as that of patients taking PPIs or in extraesophageal reflux, the gastric enzyme pepsin is active up to pH6.5 and stable up to pH8 and can be endocytosed, leading to inflammatory and carcinogenic changes in the aerodigestive tract mucosa, including in the esophagus [8,33,34,35,36].

Our group recently identified the HIV protease inhibitor, amprenavir, as a potential pepsin-targeting therapeutic for laryngopharyngeal reflux (LPR). Amprenavir inhibited pepsin at low micromolar concentrations and its prodrug fosamprenavir prevented histologic changes in an LPR mouse model [37]. In parallel work, we found that pepsin was required for esophageal and laryngeal epithelial barrier disruption at pH4 and that amprenavir preserved laryngeal epithelial integrity and prevented damage to a critical cell adhesion molecule elicited by pepsin at pH4 [38,39]. Barrier disruption is a key mechanism of GERD pathogenesis, contributing to symptom origination, sensitivity to reflux insult, immune cell infiltration, and prolonged recovery [40,41,42,43,44]. Adhesion molecules such as E-cadherin confer epithelial integrity and regulate important cellular processes such as cell proliferation, migration, and epithelial mesenchymal transition. E-cadherin damage or misexpression is associated with epithelial barrier dysfunction during GERD, progression of BE to EAC, and EAC prognosis [45,46,47,48]. This study aimed to investigate the therapeutic potential of amprenavir for GERD by investigating its protection against pepsin-induced epithelial barrier disruption and related cancer-associated molecular changes in an esophageal cell-culture model.

## 2. Results

### 2.1. Amprenavir Rescues Pepsin-Mediated Cell Dissociation

**Figure 1:**
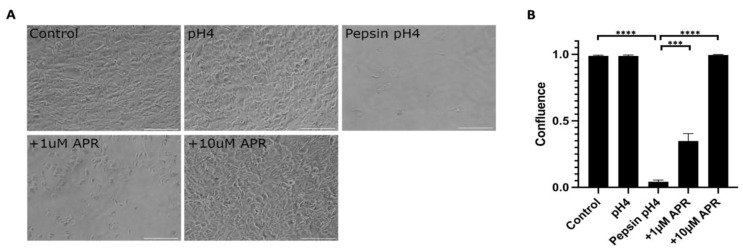
Amprenavir (10 µM) rescued cell dissociation caused by 1 mg/mL pepsin at pH4. The partial rescue was achieved by 1 µM APR. Acid alone did not cause cell dissociation. (**A**) Representative images of experiments performed in triplicate. Scale bars equal 100 µm. (**B**) Confluence as calculated using the FIJI plug-in PHANTAST (see Methods for details). Where significant, the following comparisons are shown: control vs. pH4, control vs. pepsin pH4, pepsin pH4 vs. +1 µM APR, and pepsin pH4 vs. +10 µM APR. *** = *p* < 0.001, **** = *p* < 0.0001.

Near-confluent hTERT-immortalized Barrett’s esophageal cells (BAR-T) cells were mock-treated or treated with pH4 acid only, 1 mg/mL pepsin in pH4 acid, or pepsin acid plus amprenavir at 1 or 10 µM for one hour (Figure 1, Supplemental Table S1). Acidified pepsin caused a near-complete cell dissociation (*p* < 0.0001, control versus pepsin pH4), whereas treatment with acid alone did not result in cell dissociation (*p* = 0.80, control versus pH4). Treatment with 1 µM amprenavir partially rescued pepsin-mediated cell dissociation to a mean of 34.9% confluence (*p* = 0.0008, pepsin pH4 versus +1 µM APR). The full rescue was achieved by 10 µM amprenavir treatment (*p* < 0.0001, pepsin pH4 versus +10 µM APR).

### 2.2. Amprenavir Partially Rescues Pepsin-Mediated E-Cadherin Cleavage

**Figure 2:**
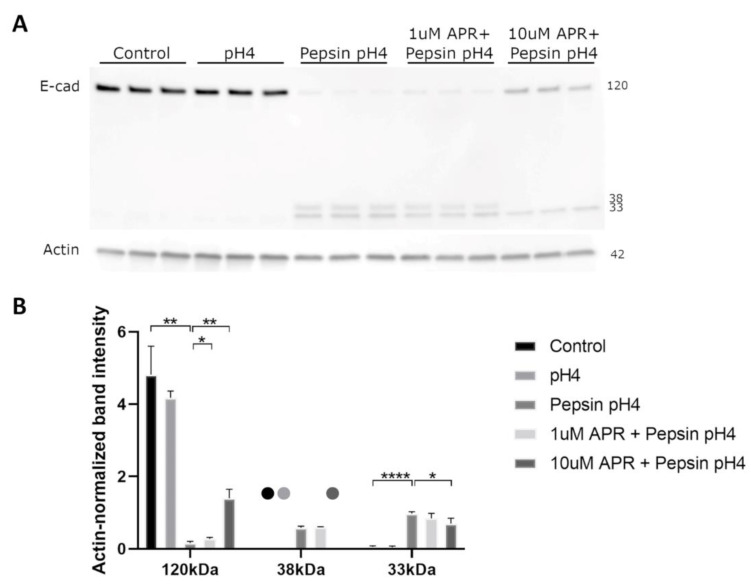
Western blot (**A**) analyzed by densitometry (**B**) showed that acidified pepsin elicited E-cadherin cleavage via regulated intramembrane proteolysis (RIP), producing C-terminal intracellular fragments of 33 and 38 kDa. Acid alone did not cause E-cadherin RIP. 10 µM amprenavir partially rescued E-cadherin RIP, with increased full-length E-cadherin and decreased 33 and 38 kDa fragments compared to acidified pepsin, whereas 1 µM amprenavir resulted in a slight increase in full-length E-cadherin only. Dots represent samples for which no bands were detected. Where significant and where bands were detectable for analysis, the following comparisons are shown: control vs. pH4, control vs. pepsin pH4, pepsin pH4 vs. +1 µM APR, and pepsin pH4 vs. +10 µM APR. * = *p* < 0.05, ** = *p* < 0.01, **** = *p* < 0.0001.

We next utilized a Western blot to investigate the ability of amprenavir to rescue E-cadherin regulated intramembrane proteolysis (RIP). Treatment of BAR-T cells with acidified pepsin, though not acid alone, resulted in the depletion of full-length E-cadherin and the production of C-terminal intracellular fragments of molecular weights consistent with E-cadherin RIP (Figure 2). Treatment with 10 µM amprenavir significantly rescued E-cadherin RIP, whereas 1 µM amprenavir resulted in a slight rescue of full-length E-cadherin cleavage though it had no statistically significant effect on C-terminal intracellular fragments (Figure 2, Supplemental Table S2). 

### 2.3. Amprenavir Protected against Pepsin-Mediated Upregulation of Matrix Metalloproteinases

**Figure 3:**
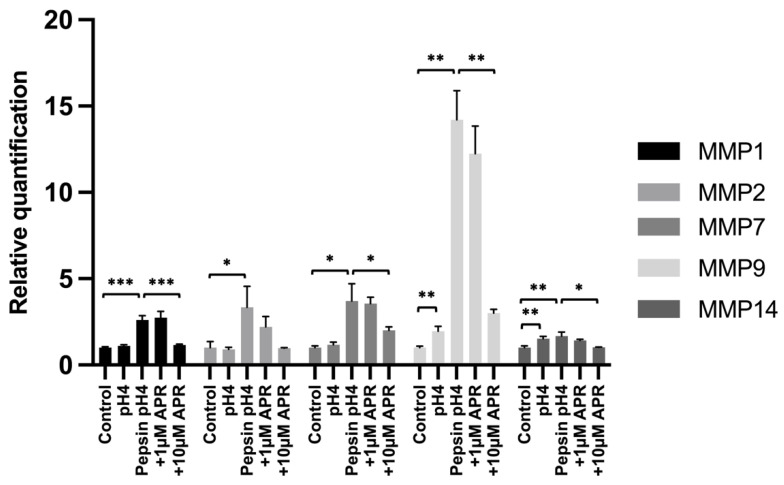
Expression of MMPs following mock treatment, treatment with acidified pepsin ±1 or 10 µM amprenavir, or acid alone. Where significant, the following comparisons are shown: control vs. pH4, control vs. pepsin pH4, pepsin pH4 vs. +1 µM APR, and pepsin pH4 vs. +10 µM APR. * = *p* < 0.05, ** = *p* < 0.01, *** = *p* < 0.001.

We next investigated the ability of amprenavir to rescue pepsin-mediated upregulation of matrix metalloproteinases (MMPs) that have been shown to be upregulated by E-cadherin fragments following RIP and/or that are known to be associated with GERD pathology. BAR-T cells were treated for 15 min, washed, and then cultured for 24 h in normal growth media prior to RNA harvest. Significant upregulation compared to an untreated control was observed following treatment with acidified pepsin for *MMP1* (*p* = 0.0004)*, MMP2* (*p* = 0.033)*, MMP7* (*p* = 0.041)*, MMP9* (*p* = 0.0052)*,* and *MMP14* (*p* = 0.0089), whereas only *MMP9* (*p* = 0.0048) and *MMP14* (*p* = 0.0056) showed statistically significant upregulation by acid alone (Figure 3, Supplemental Table S3) Treatment with 10 µM amprenavir led to statistically significant reductions in pH4 pepsin-mediated induction of *MMP1* (*p* = 0.0006), *MMP7* (*p* = 0.043), *MMP9* (*p* = 0.0066), and *MMP14* (*p* = 0.034), as well as a reduction in *MMP2* expression that did not reach statistical significance (*p* = 0.077). Treatment with 1 µM amprenavir did not significantly reduce the peptic induction of any MMP investigated. 

## 3. Discussion

The development of the PPI class of drugs represented a breakthrough in the treatment of GERD. While PPIs have provided symptom relief for millions of patients, important therapeutic challenges remain in the management of GERD. PPIs reduce the acidity of refluxate but do not target nonacid components such as pepsin and bile salts and in fact may increase their toxicity and concentration [30,31]. Ten Kate et al. found that 60 mg daily omeprazole increased pepsin in refluxate from 0.8 ± 0.1 to 2.7 ± 0.4 mg/mL (unmedicated versus omeprazole cohorts) by virtue of reducing gastric acid secretion, and thereby, volume [30]. The concentration of pepsin by PPIs may have a pathologic consequence, as indicated by experiments in an airway epithelial cell culture model which found greater inflammatory cytokine secretion, barrier disruption, and neutrophil transepithelial migration in cells exposed to gastric juice from acid-suppressed patients relative to those not taking PPIs [44]. The dose-dependent effects of pepsin have similarly been illustrated in animal and culture models of the esophagus [8,49,50].

Pepsin is found in all refluxate, and a substantial body of literature supports its contribution to reflux-attributed pathology irrespective of acid. While much of this work focused on anatomical locations proximal to the esophagus [34,51,52,53,54,55,56,57,58,59] evidence suggests that pepsin, independent of acid, elicits damage to the esophagus via similar molecular mechanisms. Pepsin harbors its greatest enzymatic activity at pH2, retains 70% of its activity up to pH6.5, and is stable up to pH8 [36]. In a nonacidic environment, pepsin is endocytosed by aerodigestive tract mucosa, including that of the larynx and esophagus. This results in cell and molecular biological changes associated with inflammation, stress, toxicity, and carcinogenesis, presumably triggered by enzymatic reactivation of pepsin in endocytic vesicles of low pH where pepsin is stored for up to 36 h following endocytosis or pepsin interaction with a receptor on the cell surface [33,34,53,59,60,61]. In alignment with an emerging model of GERD pathogenesis, mediated by inflammatory insult rather than caustic acid-induced injury [62], interleukin-8 (IL-8), a neutrophil chemoattractant and nexus of cell signaling networks regulating proliferation, apoptosis, epithelial-mesenchymal transition, and migration, appears to play a key role in the peptic injury of the larynx and esophagus [8,44,53,63,64]. IL-8 is elevated in GERD patients, highest in patients with BE and EAC, and reduced in BE patients following antireflux surgery [8,64]. In esophageal cells, chronic stimulation with nonacid pepsin elicited secretion of IL-8 and induced transition from a noncancer (*KRT10* high, *KRT8* low) to BE-associated cytokeratin profile (*KRT10* low and *KRT8* high) [8,65]. Weeks of acid pulses alone (pH 3.5 for 1 h, 3 days per week) did not incur such changes [66] in accord with poor chemoprevention by acid suppression therapy. Further, brief (5 min) exposure to pepsin with 4 or 20-h exposure to pepsin at pH7 doubled the expression of *PTGS2* (gene encoding COX-2), which is associated with chronic inflammation and cancer, and is a prognostic indicator of EAC [67,68]. COX-2 inhibitors have been shown to abrogate cell proliferation in EAC cells in vitro [69], reduce tumor incidence in animal models of EAC [70], and slow BE cell proliferation in a clinical trial [71]. Interestingly, an epidemiologic study demonstrated that long-term PPI therapy has no advantageous effect on COX-2 expression [72], suggesting that nonacid constituents of refluxate may be responsible for continued dysregulation of COX-2 in patients taking PPIs. Stable ectopic coexpression of pepsinogen and the gastric proton pump in a BE cell line also led to upregulated expression of transcripts associated with BE, EAC, and carcinogenesis including *TGFB1* and *ERBB2* [35]; pathway analysis of the global transcriptomic changes identified cancer as the top associated disease, regulation of epithelial–mesenchymal transition by growth factors as a top associated canonical pathway and cell cycle regulation, cell growth/proliferation/death/survival, DNA replication and repair, and lipid metabolism as top associated networks [35]. In contrast to the hyperproliferative effects of pepsin, acid (pH4) alone has been shown to exert an antiproliferative effect in esophageal cells [73,74].

The refluxate of untreated GERD patients is commonly acidic (63% pH < 4) with which >80% of symptom episodes are associated [75]. That of acid-suppressed GERD patients is commonly weakly to nonacidic (80% episodes pH ≥ 4) giving rise to 72% of symptom episodes [76]. In the context of acidic or weakly acidic reflux, pepsin is enzymatically active and is required for some forms of mucosal injury. For example, Tobey et al. showed that 1 mg/mL pepsin at pH1.7 caused histological damage to esophageal mucosa and irreversible loss of epithelial barrier integrity (electrical resistance) in rabbit esophagi ex vivo, whereas histological damage was absent and barrier disruption milder and reversible, provided an acid pH1.7 alone [49]. Nagahama et al. demonstrated that treatment with pepstatin, an inhibitor of pepsin, prevented esophageal lesions in a surgical rat model of reflux without altering the acidity of reflux [77]. Goldberg et al. demonstrated that acid pH > 1.3 alone caused minimal esophagitis in an in vivo cat model and that pepsin dramatically exacerbated esophagitis and histological epithelial damage at pH 1.6–2.0 in a dose-dependent manner [50]. Similarly, we recently demonstrated that weakly acidified pepsin, though not acid (pH4) alone, caused barrier dysfunction and cell detachment in a human esophageal cell culture model and that a pepsin inhibitor, sodium alginate, could prevent such damage [39]. 

Barrier disruption by pepsin is thought to be caused by damage to the protein constituents of apical junction complexes which facilitate cell–cell junctions, maintain epithelial integrity, and regulate cell proliferation and migration. Accordingly, damage to intercellular junctions was shown to precede pepsin-induced erosive lesions in the rabbit esophageal epithelium by Tobey et al. [49], and cleavage of the major protein constituent of adherens junctions, E-cadherin, is observed in GERD and LPR biopsies [45,78,79,80]. Jovov et al. demonstrated that the deletion of E-cadherin in the adult mouse esophagus was sufficient to cause barrier dysfunction, as indicated by macromolecular flux [45]. In agreement with these findings, we recently demonstrated that E-cadherin was cleaved by weakly acidic pepsin (though not acid pH4 alone) in human esophageal and laryngeal cells in vitro and that E-cadherin cleavage was rescued by pepsin inhibitors (sodium alginate in esophageal cells and amprenavir in laryngeal cells) [38,81]. The E-cadherin cleavage fragments produced by human esophageal and laryngeal epithelial cells in response to pepsin were consistent with those observed in GERD and LPR biopsies and indicative of RIP. RIP is a highly regulated and evolutionarily conserved process by which a protease (“sheddase”) cleaves an intramembrane protein near the cell surface, thereby initiating subsequent cleavage near the cytoplasmic surface by intramembrane cleavage proteases and release of cleavage fragments which harbor biological activity [82]. In the case of E-cadherin RIP, the extracellularly released fragment promotes cancer-associated changes including increased signaling through growth factor receptors (epithelial growth factor receptor and insulin-dependent growth factor receptor one), inhibition of hippo signaling and related apoptosis, immune evasion, cell migration/invasion, and transcriptional upregulation of *MMP-2*, *9*, and *14*; the intracellular domain has been implicated in Wnt/β-catenin signaling, which in turn regulates cell proliferation and migration as well as the transcription of specific MMPs [83,84,85]. MMPs are a family of enzymes that weaken epithelial integrity by degrading extracellular matrix components. Their upregulation by E-cadherin RIP fragments is especially notable given that several MMPs are associated with GERD, its severity, and EAC, and have known roles in carcinogenesis [86,87,88,89]. 

Herein, the treatment of esophageal cells with acidified pepsin, though not acid alone (pH4), caused near complete E-cadherin RIP within 30 min and cell dissociation in one hour. Prior work has indicated that E-cadherin RIP fragments elicit transcription of *MMP-2*, *9*, and *14* 24 h poststimulation in lung cells [83]. Herein, *MMP-2*, *9*, and *14* were elevated at 24 h postexposure to pepsin acid. This suggests that E-cadherin RIP fragments produced by esophageal cells in response to pepsin-acid stimulation are biologically active and exert carcinogenic changes consistent with their function in other cell types. Importantly, the pepsin inhibitor, amprenavir, attenuated or rescued these effects and provided concentrations found in the serum of patients taking the manufacturer-recommended dose of fosamprenavir for HIV [90]. These data are in alignment with prior work demonstrating that the pepsin inhibitor, pepstatin, prevents esophageal lesions in a surgical rat model of GERD [77] and that the pepsin inhibitor, sodium alginate, prevents esophageal and laryngeal epithelial barrier disruption by pepsin pH4 [39]. Pepsin-acid-induced E-cadherin RIP and protection by amprenavir appear to be conserved across cell types. In a recently submitted work, we found that amprenavir abrogates epithelial cell disruption, E-cadherin cleavage, and MMP dysregulation by pepsin pH4 in laryngeal cells, supporting the potential utility of amprenavir for both GERD and LPR [38]. Given that amprenavir protects epithelial barrier integrity, which is, in turn, a facet of GERD symptom origination, the data herein suggest that amprenavir may provide relief for the 20–40% of GERD patients with symptoms recalcitrant to PPI therapy [91,92,93]. Further, the capacity of amprenavir to rescue a pepsin-induced proinvasive cell phenotype characterized by E-cadherin RIP and MMP induction supports its chemopreventive potential. This is particularly intriguing in light of the failure of PPIs to demonstrate chemopreventive benefits for patients with GERD as well as burgeoning evidence that pepsin promotes carcinogenic changes in the esophagus [23,24,30,31,35,53,57,61]. Future in vitro work is warranted to address amprenavir protection against additional pepsin-induced molecular changes harboring a strong correlation with GERD severity and/or EAC, such as IL-8 and PTGS2/COX-2, and cancer-related cellular processes known to be impacted by pepsin such as cell-cycle regulation. Corroboration of the chemopreventive benefits of amprenavir is warranted in in vivo models of BE and EAC.

In summary, our results demonstrate that amprenavir, at serum concentrations achievable using the manufacturer-recommended dose of fosamprenavir, protects against esophageal epithelial barrier disruption and contributes to molecular mechanisms in a cell culture model of weakly acid reflux. Further, these data suggest that amprenavir may provide chemopreventive benefits. This work has important implications for the treatment of PPI-recalcitrant GERD, which affects a large number of patients, and for the prevention of EAC which is increasing at an alarming rate and is poorly addressed by current first-line therapy for GERD. This work supports the potential benefit of pepsin-targeting therapeutics as adjunctive therapies for GERD and an upcoming randomized clinical trial planned to assess the efficacy of amprenavir for the prevention of signs and symptoms of GERD. 

## 4. Materials and Methods

### 4.1. Cell Culture and Treatment

hTERT-immortalized Barrett’s esophageal cells BAR-T [94] (the kind gift of Rhonda F. Souza) were cultured as previously described [39] to 90% confluence for examining protein expression and 50% for gene expression. 

The IC50 of amprenavir for porcine pepsin (3.56 uM) was determined in a prior study [37]. The dose for study herein was selected based upon the serum concentration observed in patients taking the manufacturer-recommended dose for the treatment of HIV [90].

In triplicate, unless noted, cultures were treated in HBSS or HBSS pH4 ±1 mg/mL porcine pepsin (Sigma-Aldrich, St Louis, MO, USA) ±1–10 uM amprenavir (APR; Sigma Aldrich) at 37 °C/5% CO_2_ for 30 min (Western blot). To measure cell dissociation, cells were treated for 75 min and washed in HBSS. For MMP qPCR, cells were treated for 15 min, washed twice in HBSS, and incubated in normal growth media at 37 °C/5% CO_2_ for 24 h prior to harvest.

### 4.2. Cell Dissociation

Images were obtained on an inverted microscope (Nikon, Tokyo, Japan and Metamorph Inc., Nashville, TN, USA). The percent cell-free area of a single image from each well (*n* = 3) was quantified using the PHANTAST plug-in for FIJI [95,96].

### 4.3. Western Blot

Treatment solutions were collected and 2 uM NaOH and protease inhibitor cocktail (ThermoFisher Scientific, Waltham, MA, USA) added. Cells were harvested in cold RIPA lysis buffer (1% NP40, 0.5% sodium deoxycholate, 0.1% SDS, 150 mM NaCl, 50 mM Tris-Cl, pH7.4) containing protease inhibitor. Proteins were separated (4–20% TGX, Bio Rad Laboratories, Hercules, CA, USA) and transferred to polyvinylidene difluoride. Membranes were blocked (5% milk, 0.1% Tween-20, PBS) and probed with: E-cadherin C-terminal (4A2C7; ThermoFisher), actin (CP01, Sigma-Aldrich), and HRP-conjugated secondary (Agilent Technologies, Santa Clara, CA, USA). Densitometry was performed using Image J (version 1.52a, National Institutes of Health, Bethesda, MD, USA).

### 4.4. Real Time qPCR

RNA was extracted via TRIZOL (ThermoFisher Scientific) and the purity and concentration were assessed by UV spectroscopy. RNA was reverse transcribed using Superscript IV VILO (ThermoFisher Scientific). The qPCR was performed in quadruplicate reactions using Taqman gene expression assays in a Viia7 instrument (ThermoFisher Scientific) per the manufacturer’s instructions. Threshold cycle (Ct) values < 36 were used for analysis, and gene expression was normalized to the housekeeping gene *HPRT1*. 

### 4.5. Statistical Analysis

Student’s *t*-test was used to compare groups. For qPCR, RQs were calculated via the delta-delta method, and *p* < 0.05 was considered significant.

## Data Availability

The data presented in this study are available herein and in the Supplementary Materials.

## Supplementary Materials

The supporting information can be downloaded at: https://www.mdpi.com/article/10.3390/ijms24076765/s1.
