# Supplementary material for: The Protease Inhibitor Amprenavir Protects against Pepsin-Induced Esophageal Epithelial Barrier Disruption and Cancer-Associated Changes
Source: Int J Mol Sci. 2023 Apr 5;24(7):6765. doi: 10.3390/ijms24076765 (PMC10095080; doi:10.3390/ijms24076765)
Supplement: Supplementary file 1 [file ijms-24-06765-s001.zip › ijms-2303782-supplementary.pdf]

Blaine-Sauer, et al. *The protease inhibitor amprenavir protects against pepsin-induced esophageal epithelial barrier disruption and cancer-associated changes*

SUPPORTING INFORMATION

Table S1. Cell confluence following mock treatment, treatment with acidified pepsin ±1 or 10µM amprenavir, or acid alone.

Cell confluence	Mean RQ±SD	p rel Control	p rel Pepsin pH4
Control	0.989±0.005		
pH4	0.988±0.007	0.80	<0.0001
Pepsin pH4	0.043±0.012	<0.0001	
Pepsin pH4 + 1µM APR	0.349±0.056	0.0024	0.0008
Pepsin pH4 + 10µM APR	0.995±0.004	0.17	<0.0001

Table S2. Actin-normalized band intensity of full-length E-cadherin and fragments following mock treatment, treatment with acidified pepsin ±1 or 10µM amprenavir, or acid alone.

	Mean RQ±SD	p rel Control	p rel Pepsin pH4
120 kDa			
Control	4.816±0.785		
pH4	4.180±0.183	0.24	
Pepsin pH4	0.165±0.041	0.0092	
Pepsin pH4 + 1µM APR	0.279±0.038		0.025
Pepsin pH4 + 10µM APR	1.402±0.246		0.0010
38 kDa			
Control	Not detected		
pH4	Not detected	N/A	
Pepsin pH4	0.584±0.045	N/A	
Pepsin pH4 + 1µM APR	0.591±0.019		0.82
Pepsin pH4 + 10µM APR	Not detected		N/A
33 kDa			
Control	0.055±0.025		
pH4	0.040±0.038	0.60	
Pepsin pH4	0.976±0.050	<0.0001	
Pepsin pH4 + 1µM APR	0.840±0.143		0.19
Pepsin pH4 + 10µM APR	0.697±0.150		0.038

Table S3. Expression of MMPs following mock treatment, treatment with acidified pepsin ±1 or 10µM amprenavir, or acid alone.

	Mean RQ±SD	p rel Control	p rel Pepsin pH4
MMP1			
Control	1.00±0.05		
pH4	1.11±0.07	0.091	0.0006
Pepsin pH4	2.61±0.25	0.0004	
Pepsin pH4 + 1µM APR	2.74±0.36	0.013	0.63
Pepsin pH4 + 10µM APR	1.15±0.06	0.028	0.0006
MMP2			
Control	1.00±0.36		
pH4	0.90±0.13	0.68	0.072
Pepsin pH4	3.34±1.21	0.033	
Pepsin pH4 + 1µM APR	2.21±0.60	0.040	0.22
Pepsin pH4 + 10µM APR	0.97±0.04	0.89	0.077
MMP7			
Control	1.00±0.10		
pH4	1.17±0.16	0.18	0.045
Pepsin pH4	3.71±0.99	0.041	
Pepsin pH4 + 1µM APR	3.55±0.37	0.0003	0.81
Pepsin pH4 + 10µM APR	2.00±0.21	0.0018	0.043
MMP9			
Control	1.00±0.09		
pH4	1.96±0.28	0.0048	0.0002
Pepsin pH4	14.21±1.68	0.0052	
Pepsin pH4 + 1µM APR	12.24±1.60	0.0065	0.21
Pepsin pH4 + 10µM APR	3.00±0.22	0.0001	0.0066
MMP14			
Control	1.00±0.11		
pH4	1.53±0.13	0.0056	0.38
Pepsin pH4	1.68±0.22	0.0089	
Pepsin pH4 + 1µM APR	1.42±0.07	0.0053	0.12
Pepsin pH4 + 10µM APR	1.02 ±0.02	0.83	0.034
